# Comparison of Salbutamol Delivery Efficiency for Jet versus Mesh Nebulizer Using Mice

**DOI:** 10.3390/pharmaceutics11040192

**Published:** 2019-04-19

**Authors:** Kyung Hwa Chang, Sang-Hyub Moon, Jin Young Oh, Young-Soon Yoon, Namyi Gu, Chi-Yeon Lim, Bong Joo Park, Ki Chang Nam

**Affiliations:** 1 Department of Medical Engineering, Dongguk University College of Medicine, Goyang-si, Gyeonggi-do 10326, Korea; chang9429@hotmail.com (K.H.C.); shnoom@naver.com (S.-H.M.); 2 Department of Internal Medicine, Dongguk University Ilsan Hospital, Goyang-si, Gyeonggi-do 10326, Korea; indr71@hanmail.net (J.Y.O.); ysyoonmd@gmail.com (Y.-S.Y.); 3 Department of Clinical Pharmacology and Therapeutics, Dongguk University Ilsan Hospital, Goyang-si, Gyeonggi-do 10326, Korea; namyi.gu@gmail.com; 4 Department of Biostatistics, Dongguk University College of Medicine, Goyang-si, Gyeonggi-do 10326, Korea; rachun@hanmail.net; 5 Department of Electrical Biological Physics and Institute of Biomaterials, Kwangwoon University, Seoul 01897, Korea

**Keywords:** nebulizer, drug delivery efficiency, salbutamol, aerosol size, residual volume

## Abstract

Recent reports using a breathing simulator system have suggested that mesh nebulizers provide more effective medication delivery than jet nebulizers. In this study, the performances of jet and mesh nebulizers were evaluated by comparing their aerosol drug delivery efficiencies in mice. We compared four home nebulizers: two jet nebulizers (PARI BOY SX with red and blue nozzles), a static mesh nebulizer (NE-U22), and a vibrating mesh nebulizer (NE-SM1). After mice were exposed to salbutamol aerosol, the levels of salbutamol in serum and lung were estimated by enzyme linked immunoassay (ELISA). The residual volume of salbutamol was the largest at 34.6% in PARI BOY SX, while the values for NE-U22 and NE-SM1 mesh nebulizers were each less than 1%. The salbutamol delivery efficiencies of NE-U22 and NE-SM1 were higher than that of PARI BOY SX, as the total delivered amounts of lung and serum were 39.9% and 141.7% as compared to PARI BOY SX, respectively. The delivery efficiency of the mesh nebulizer was better than that of the jet nebulizer. Although the jet nebulizer can generate smaller aerosol particles than the mesh nebulizer used in this study, the output rate of the jet nebulizer is low, resulting in lower salbutamol delivery efficiency. Therefore, clinical validation of the drug delivery efficiency according to nebulizer type is necessary to avoid overdose and reduced drug wastage.

## 1. Introduction

Nebulizers are commonly used to treat and manage a variety of diseases, including asthma, chronic obstructive pulmonary disease (COPD), cystic fibrosis, and pneumonia. Nebulizers are medical devices that generate an aerosol by agitating a medication solution and deliver drugs rapidly and directly into the airways. Nebulizers have many advantages compared to systemic delivery, such as the ability to use a smaller dose, faster onset of the effects, topical application of a drug to the lungs, fewer systemic side effects, and ease and convenience to the patient [1].

Various types of nebulizers have been developed to improve the efficiency of drug delivery to patients, and these can be classified into three types according to their working principle: jet, ultrasonic, and mesh nebulizers. Jet nebulizers are generally considered the gold standard method in the clinical field for delivering medicines to patients requiring aerosol therapy. However, compressor-based jet nebulizer systems are inconvenient because they require extra tubing, their associated treatment times are long, the compressors are heavy and generate noise during their operation, and mechanical shear forces can affect certain medications [2,3,4]. It has been reported that jet nebulizers show wide variations in flow rate and aerosol mass distribution depending on the specific nebulizer/compressor combinations, and they can have a large residual volume in the reservoir due to incomplete nebulization of the medication [5,6,7]. Jet nebulizers are inefficient in drug delivery because of the variability of the actual dose delivered to the airways and lungs. 

In order to overcome many of the limitations associated with jet nebulizers, new mesh type nebulizers have been developed, which force liquid medications through multiple apertures in a mesh or aperture plate to generate aerosol. Mesh nebulizers are small and portable due to their use of batteries, and they are noise free during operation, with fast treatment times and higher output efficiency [8,9]. In addition, mesh nebulizers produce consistent and improved aerosol, resulting in a predominantly fine-particle fraction reaching into the peripheral lung, and they have minimal residual volume and the ability to nebulize in low drug volumes. These major advantages of mesh nebulizers increase drug delivery efficiency when using antibiotic solutions such as tobramycin and amikacin [10,11]. Mesh nebulizers can be divided into two types depending on their aerosol production principle: static (passive) and vibrating (active). Static mesh nebulizers use a horn vibrating ultrasonically against a static mesh, while vibrating mesh nebulizers use a mesh mounted in an ultrasonically vibrating piezo ring [3,8,12]. The vibrating mesh nebulizer was also reported for its suitability in achieving an appropriate in vitro inhalation performance model [13].

According to recent reports, the drug delivery efficiency of mesh nebulizers was generally higher than that of jet nebulizers in studies using breathing simulator modules in both pediatric and adult settings [13,14,15,16,17]. In addition, the aerosols delivered by mesh nebulizers provided more effective medication deposition in the lung and blood as compared to jet nebulizers in studies using radio-tagged aerosols (99mTc diethylenetriamine pentaacetate: 99mTc-DTPA) and antibiotics in macaques, cystic fibrosis patients and healthy humans [10,18,19]. Therefore, clinicians have increasingly focused on how certain nebulizers will perform in administering certain drugs to ensure clinical suitability. 

Glucocorticoids, bronchodilators, antibiotics and mucus hydrates have mainly been used for studies on nebulizer performance and drug delivery efficiency in vitro and in vivo. Among them, salbutamol (also known as albuterol, and marketed as Ventolin) is a bronchodilator commonly used in the home and hospital to prevent and treat difficulty breathing, wheezing, and shortness of breath caused by asthma and COPD. However, comparative studies on salbutamol delivery efficiency in various types of nebulizers have mostly been based on the use of a breathing simulator with mechanical ventilation, a humidifier, and breathing masks [14,16]. A few clinical studies have compared the efficacy, admission rates, and total dose of salbutamol used by jet and mesh nebulizers in asthmatic children and emergency rooms [20,21].

The purpose of this study is to investigate the performance of and in vivo salbutamol delivery efficiency on four commonly-used home nebulizers: two jet nebulizers (PARI BOY SX with red and blue nozzles), a static mesh nebulizer (NE-U22), and a vibrating mesh nebulizer (NE-SM1). The salbutamol delivery efficiency was evaluated on spontaneously breathing mice without mechanical ventilation and breathing masks in a closed inhalation chamber.

## 2. Materials and Methods 

### 2.1. Materials and Nebulizers

Salbutamol (Ventolin respirator solution, 5 mg/mL salbutamol sulfate) was acquired from GlaxoSmithKline (Brentford, UK). In this study, 0.5 mL of the respirator solution was diluted with 1.5 mL of sterile normal saline solution, resulting in 1.25 mg/mL salbutamol. The competitive enzyme linked immunoassay (ELISA) kit for salbutamol was purchased from Bio Scientific Corporation (MaxSignal^®^, Austin, TX, USA). Indocyanine green (ICG) was obtained from BioActs (Incheon, Korea). Four home nebulizers were investigated in this study, and the operating modes and abbreviations of each nebulizer are presented in Table 1. In this study, PARI BOY SX was used with two types of nozzles, red and blue, which were supported by the manufacturer. According to the manufacturer, the red nozzle of PARI BOY SX creates an even finer aerosol than the blue nozzle.

### 2.2. Animals

All animal experiments were conducted in accordance with policies approved by the Ethics Committee of Animal Service Center at Dongguk University (IACUC Number: 2016-013-1, 4 April 2016). Female BALB/c mice aged seven to eight weeks were obtained from Daehan Biolink (Eumseong, Korea). The mice were housed in standard cages and maintained under controlled constant room temperature and humidity with a 12-h light/dark cycle. The animals had free access to both water and food.

### 2.3. Size of Nebulized Aerosol Droplets

The aerosol size distribution for each nebulizer was assessed using a Spraytec (Malvern instrument, Malvern, UK) which was analyzed by the laser diffraction method and is a very reliable and time-saving method [22]. The 0.1% salbutamol was used as the test formulation according to the International Organization for Standardization (ISO) 27427:2013 (E) that deals with specification requirements for the safety and performance testing of general-purpose nebulizing systems [23]. In order to prepare 0.1% salbutamol, 0.1 g of salbutamol was dissolved in 100 mL of saline (1 mg/mL). The nebulized aerosol size of 1 or 1.25 mg/mL salbutamol was measured during nebulization for 1 min in each of the four nebulizers after being filled with 2 mL of analytical solution. The fifty percent volume diameter (Dv(50)) results were automatically calculated by the Spraytec software (version 3.1, Malvern instrument, Malvern, UK, 2009). This process was repeated 3 times for each nebulizer.

### 2.4. Residual Volume, Nebulization Time and Output Rate in Each Nebulizer

To estimate the residual volume, nebulization time, and output rate, all nebulizers were operated in accordance with the manufacturer’s recommendations. After filling the reservoir of each nebulizer with 2 mL of 1 or 1.25 mg/mL salbutamol, the nebulizers were operated for the duration of nebulization (until the onset of sputter or until dryness). The nebulizer weight was measured before and after nebulization, and the nebulization time was also recorded. The residual volume was determined gravimetrically, and the evaporation was negligible [24]. The output rate (OR) was calculated according to the equation below. This process was repeated three times for each nebulizer.

$$Output\;Rate\;\left(OR\right)\;\left(mL/min\right)=\frac{charged\;volume\;\left(mL\right)-residual\;volume\left(mL\right)}{duration\;of\;nebulization\;\left(min\right)}$$

### 2.5. Exposure of Salbutamol to Mice

**Figure 1:**
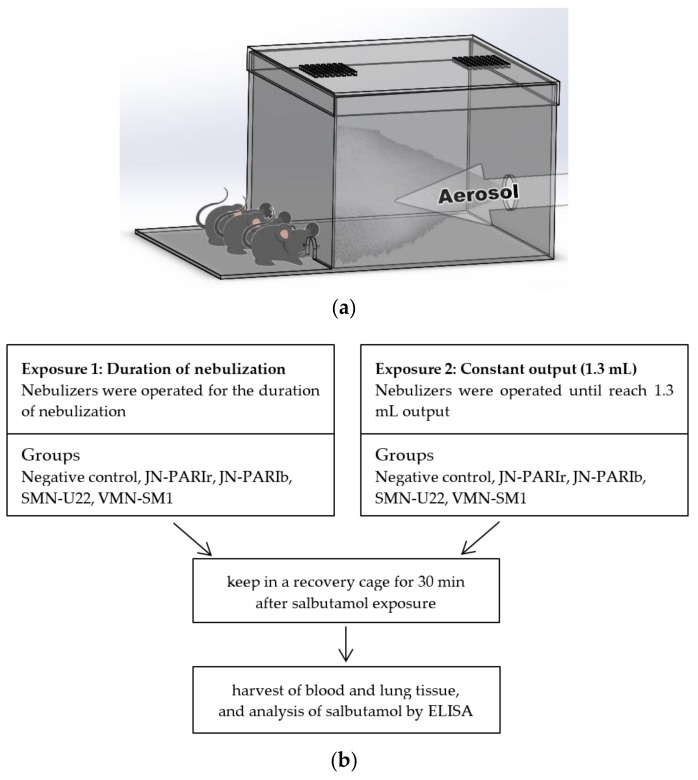
Graphic description of the head-only inhalation exposure system and experimental procedure: (**a**) the nebulizer was activated at the opposite side to the mice heads; (**b**) the delivery efficiencies of salbutamol in the four nebulizers were examined under two exposure conditions. Blood and lung tissue were harvested from salbutamol-exposed mice, and further used for enzyme linked immunoassay (ELISA).

The mice were exposed to salbutamol (1.25 mg/mL) in a closed inhalation exposure chamber (Figure 1a) that was designed specifically for exposure to three mice at once. The chamber consisted of clear acrylic sheets (3 mm in thickness) with external dimensions of 20 × 20 × 15 cm and internal volume of 6 L, and three small holes for head-only exposure. The mice were randomly divided into five groups (*n* = 3 mice/group) as follows: (i) untreated (negative control); (ii) nebulized by JN-PARIr; (iii) nebulized by JN-PARIb; (iv) nebulized by SMN-U22; and (v) nebulized by VMN-SM1. The mice were anesthetized via intraperitoneal injections of ketamine (100 mg/kg) and xylazine (10 mg/kg) prior to undergoing nebulization. The salbutamol delivery efficiencies in four nebulizers were examined under two different exposure conditions: 1) 2 mL of salbutamol was placed in the reservoir, then the nebulizers were operated for the duration of nebulization (until the onset of sputter or until dryness), and 2) 2 mL of salbutamol was placed in the reservoir, then the nebulizer was run until the output volume of salbutamol reached 1.3 mL (Figure 1b). Following salbutamol exposure, the mice were kept in a recovery cage for 30 min, and then sacrificed by CO_2_ asphyxiation to analyze for salbutamol levels. The remaining amount of the drug in the chamber was not measured; but in humans, inhalation of the drug only reaches 10–20% of the deeper portion of the bronchi.

### 2.6. Blood and Tissue Collection

Blood was collected from abdominal aorta in an untreated test tube, then incubated to clot at room temperature for 1 h. To separate serum, the collected blood was centrifuged at 2000× *g* for 10 min to remove clots, and then the serum was aliquoted into fresh micro-tubes. Whole lung tissues were harvested to one clean microtube after being perfused with cold phosphate buffered saline (PBS) to remove all red blood cells. The serum and lung tissue were kept at −80 °C until further analysis.

### 2.7. Determination of the Level of Salbutamol in Serum and Lung Tissues

The quantitative assessment of salbutamol in serum and lung tissue was performed using a Salbutamol ELISA Test Kit (MaxSignal^®^). The ELISA was performed according to the manufacturer’s instructions. In this study, the Salbutamol ELISA Test Kit was not conducted on matrix-based standard curves and quality control using a minimum of six standard points, excluding blank. Only the spiking experiment using 3 concentrations (0.15, 1.5 and 4.5 ng/mL) of salbutamol were performed in serum, and the recovery (%) of spiked salbutamol was between 95–100%. The serum from salbutamol-exposed mice by JN-PARIr and JN-PARIb were diluted 5-fold, and the serum from salbutamol-exposed mice by SMN-U22 and VMN-SM1 were diluted 20-fold in 1× PBS buffer provided in the ELISA kit. Frozen whole lung tissues were thawed and added with 0.5 mL of cold Hank’s Balanced Salt Solution (HBSS). The whole lung tissues were homogenized using T 10 basic ULTRA-TURRAX^®^ (IKA, Staufen, Germany) for 1 min at level 5 on ice. After homogenization, lung homogenates were centrifuged at 16,000× *g* for 10 min at 4 °C, and the supernatants were collected for ELISA analysis. The supernatants were diluted 5-fold in sample buffer provided with the ELISA kit. The optical density (OD) values of the samples were determined at 450 nm using a SpectraMax M3 microplate reader (Molecular Devices, Sunnyvale, CA, USA). The concentrations of salbutamol were read from a six-point calibration curve, and further multiplied by the corresponding dilution factor. 

### 2.8. In Vivo Lung Imaging Using ICG Nebulization

ICG was initially dissolved in water at a concentration of 1 mg/mL, and this stock solution was further diluted with saline to a final concentration of 25 μg/mL. Mice were anesthetized and placed in an inhalation exposure chamber. The mice were exposed to ICG (25 μg/mL) until the output volume reached 1.3 mL using JN-PARIb and VMN-SM1. Following ICG exposure, lung tissues were immediately harvested from mice, then placed into the imaging chamber. Lungs were aligned to the field of view of the fluorescence-labeled organism bioimaging instrument (FOBI) in an in vivo Imaging System (NeoSciences Co. Ltd., Suwon, Korea), and the near infrared channel was used for ICG imaging. The integrated density of the ICG fluorescence signal was calculated using NEOimage software (version 3.3, NeoSciences Co. Ltd., Suwon, Korea, 2016). The lung tissues were transferred into amber microcentrifuge tubes after imaging with FOBI and kept at −80 °C until further analysis for fluorescence intensity in lung homogenates. Frozen lung tissues were thawed and added with 0.5 mL of cold HBSS, then homogenized using T 10 basic ULTRA-TURRAX^®^ for 1 min at level 5 on ice. Lung homogenates were centrifuged at 16,000× *g* for 10 min at 4 °C, and then 200 μL of supernatants was transferred to a 96-well black plate. Fluorescence intensity at Ex/Em = 780/830 nm was measured using a Spark 10M multimode reader (Tecan, Zurich, Switzerland). 

### 2.9. Statistical Analyses

The means and standard deviations (SD) were calculated for all experimental groups. Data were subjected to one-way analysis of variance followed by Dunn’s test to determine whether the differences relative to the PARIr or PARIb group were significant. Statistical analysis was performed using SigmaPlot software version 13 (Systat Software, San Jose, CA, USA, 2014). *p* values of <0.05 were considered to be statistically significant.

## 3. Results

### 3.1. Measurement of Particle Size and Performance of Nebulizer

First, to understand the differences in functional performance among the four home nebulizers, we compared the mass median diameter (MMD) of aerosol particle sizes of 1 and 1.25 mg/mL salbutamol. In order to measure the aerosol particle size, all nebulizers were placed into Spraytec without mask adapters and mouthpieces. The Dv(50) results for 1 mg/mL salbutamol were very similar to those for salbutamol (1.25 mg/mL) (Table 2). The Dv(50) results for 1 mg/mL salbutamol, using JN-PARIr and JN-PARIb, were differentially measured according to their nozzle type: 3.40 ± 0.09 μm for the red nozzle and 4.54 ± 0.03 μm for the blue nozzle. The Dv(50) results for 1 mg/mL salbutamol, using SMN-U22 and VMN-SM1, were 6.83 ± 0.09 μm and 5.34 ± 0.18 μm, respectively. The aerosol particle sizes by JN-PARIr were measured as the smallest among the tested nebulizers for all tested solutions, while those by SMN-U22 were the largest.

Next, we examined the residual volume, nebulization time, and output rate after filling each reservoir with 2 mL of 1 and 1.25 mg/mL salbutamol (Table 3). The results for 1 mg/mL salbutamol were comparable to those for 1.25 mg/mL salbutamol. JN-PARIr and JN-PARIb stopped working when the sputter sound occurred. The residual volumes of 1.25 mg/mL salbutamol were approximately 27% for JN-PARIr and 34.6% for JN-PARIb, while those for both SMN-U22 and VMN-SM1 were small enough to be negligible. The nebulization times of SMN-U22 for 1 and 1.25 mg/mL salbutamol were shorter than those of other nebulizers, 6.48 ± 0.03 min and 6.50 ± 0.09 min, respectively, while JN-PARIr showed the longest nebulization time, 9.24 ± 0.05 min for 1 mg/mL salbutamol and 9.33 ± 0.08 min for 1.25 mg/mL salbutamol. Upon completion of nebulization, the output rate of each nebulizer was calculated. The output rates of 1.25 mg/mL salbutamol in JN-PARIr, JN-PARIb, SMN-NE1, and VMN-U22 were 0.153 ± 0.004, 0.184 ± 0.002, 0.288 ± 0.006, and 0.239 ± 0.003 mL/min, respectively. The output rate of SMN-U22 was the highest, while that of JN-PARIr was the lowest; these results were the same for the aerosol particle size. 

### 3.2. Delivery Efficiency of Salbutamol in Mice

To evaluate the delivery efficiencies of the four nebulizers, mice were exposed to salbutamol (1.25 mg/mL) using a closed inhalation exposure chamber, then the salbutamol level in serum and lung homogenates was quantified using ELISA. Salbutamol was nebulized under two different exposure conditions. The first exposure condition (Exposure 1) is the common protocol in clinical use. First, 2 mL of salbutamol was placed in the reservoir and then the nebulizers were operated for the duration of nebulization (until the onset of sputter or until dryness). In this condition, the outputs from the four nebulizers were different, resulting in different residual volumes, as shown in Table 3. The second exposure condition (Exposure 2) set the output to 1.3 mL on all four nebulizers (Table 4). Since the residual volume of JN-PARIb was approximately 0.7 mL from 2 mL of salbutamol, 1.3 mL was determined as the constant output.

**Figure 2:**
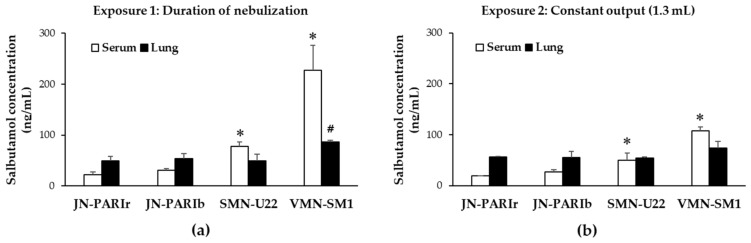
Salbutamol level in serum and lung tissues of mice exposed to four nebulizers: (**a**) After 2 mL of salbutamol was released in the reservoir, then the nebulizers operated for the duration of nebulization (Exposure 1) (*n* = 9 mice/group); (**b**) Nebulizers operated until reaching 1.3 mL of output (Exposure 2) (*n* = 9 mice/group). All values are means ± SD. * *p* < 0.05 versus serum of JN-PARIr, # *p* < 0.05 versus lung of JN-PARIr.

As shown in Figure 2a,b, the SMN-U22 and VMN-SM1 groups show significantly higher levels of salbutamol in serum than the JN-PARIr group under Exposure 1 (JN-PARIr: 22.1, JN-PARIb: 31.1, SMN-U22: 78.1, VMN-SM1: 227.2 ng/mL) and Exposure 2 (JN-PARIr: 18.9, JN-PARIb: 27.5, SMN-U22: 50.2, VMN-SM1: 107.6 ng/mL). No significant difference was found in the serum levels of salbutamol between the JN-PARIr and JN-PARIb groups in both exposure conditions. In Exposure 1, the serum salbutamol levels of the SMN-U22 and VMN-SM1 groups were generally higher than those of Exposure 2, while those of JN-PARIr and JN-PARIb were not substantially different. There was no statistically significant difference in the salbutamol concentrations of the lung tissues, except for VMN-SM1 in Exposure 1.

### 3.3. ICG Delivery in Mice Lung Tissue

Fluorecence imaging was used to investigate whether the mesh nebulizer had better drug delivery efficiency than the jet nebulizer. ICG dye is used as an indicator substance in preclinical and clinical applications [25,26]. Also, that it is possible to monitor the delivery and consumption of ICG after ICG nebulization in mice has been previously suggested [27,28]. JN-PARIb and VMN-SM1 were selected among four nebulizers. As shown in Table 2, the difference in Dv(50) results between JN-PARIb and VMN-SM1 was less than 1 μm, and this could minimize the effect of aerosol particle size on drug delivery efficiency [29]. The salbutamol concentration at 30 min after exposure was not statistically significantly different in lung tissue except with VMN-SM1 at Exposure 1. Therefore, we attempted to visualize the remaining amount of ICG in the lungs before complete systemic circulation immediately after exposure to ICG.

**Figure 3:**
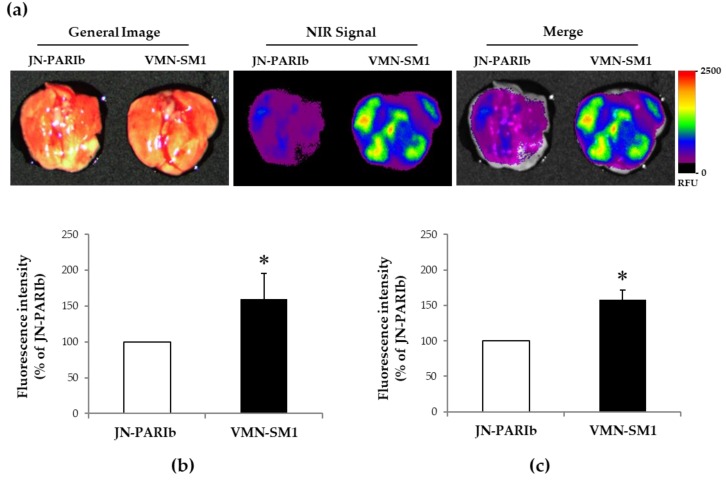
Representative fluorescence images of lung tissues: (**a**) The representative excised lung tissues from ICG-exposed mice by JN-PARIb and VMN-SM1 were assessed for ICG fluorescence intensity using a color scale to indicate relative fluorescence units (RFU) (left to right; General Image, NIR signal, Merge); (**b**) Relative fluorescence intensity of ICG signals from lung tissues imaging (*n* = 18 mice/group); (**c**) Relative fluorescence intensity from lung homogenates of ICG-exposed mice (*n* = 9 mice/group). Values are means ± SD. * *p* < 0.05 versus JN-PARIb.

The fluorescence signal from the control lung tissues was not detectable (data not shown). As shown in Figure 3a, the ICG fluorescence signal was higher in the VMN-SM1 than the JN-PARIb group. The integrated intensity of lung tissues from ICG-exposed mice using VMN-SM1 was 60% higher than in those using JN-PARIb (Figure 3b). As a result of the ICG integrated intensity in lung tissues, lung homogenates of ICS-exposed mice using VMN-SM1 showed an increase in fluorescence intensity compared with JN-PARIb (Figure 3c). These results support that the levels of salbutamol in the serum were significantly higher in the VMN-SM1 groups than in the JN-PARIr group after exposure to salbutamol under the Exposure 2 (Figure 2b).

## 4. Discussion

Aerosol therapy via a nebulizer is mainly used to relieve the respiratory symptoms of children and adults in the home and hospital. Nebulizers vary in their output characteristics and dosing rates depending on their working principles. Particle size and output rate are critical factors in determining the total and regional lung deposition of inhaled aerosols [29]. In this study, the performance and in vivo salbutamol delivery efficiencies of four home nebulizers, JN-PARIr, JN-PARIb, SMN-U22 and VMN-NE1, were compared. The aerosol particle sizes of the jet nebulizers were generally smaller than those of the mesh nebulizers. The jet nebulizers were able to produce different aerosol particles in the same nebulizer by changing the nozzle type. This is a unique feature of the jet nebulizer, and the size of aerosol particles produced by the jet nebulizer is directly proportional to the size of the nozzle, which can generate much smaller particle sizes [30]. The aerosol sizes for JN-PARIr, JN-PARIb and SMN-U22 were larger than the manufacturer specifications except for VMN-SM1. The manufacturer specifications measured aerosol particle size by mass median aerodynamic diameter (MMAD), which captured aerosol particles in a cascade impactor driven by air [23], and while MMD was measured by the laser diffraction method in this study. According to performance data, the residual volumes of JN-PARIr and JN-PARIb were significantly larger than those of SMN-U22 and VMN-SM1, which means lower there was a lower output of the latter two. The nebulization times of JN-PARIr and JN-PARIb were generally longer than those of the mesh nebulizers, resulting in lower output rates. This result is consistent with others of previous studies for the performance comparison of jet and mesh nebulizers [5,6,7]. 

Since the measurement of the in vitro drug delivery efficiency of the nebulizers should be as realistic as possible, a mechanical model has been developed that can simulate in vivo breathing [31,32,33]. However, a breathing simulator cannot completely replace in vivo evaluations, due to the limitation of the simulator operation, so an effective in vivo model is needed to evaluate the drug delivery efficiency of the nebulizer [34]. Generally, breathing simulator models use mechanical ventilation to compare the drug delivery efficacy of mesh nebulizers and jet nebulizers. However, we focused on the use of a mild patient, who needed a nebulizer to treat their disease at home without mechanical ventilation. Thus, the mice were exposed to salbutamol under spontaneous breathing after anesthesia without mechanical ventilation and breathing masks. Anesthesia can reduce their respiratory functions such as respiratory rate or tidal volume compared to normal mice. Jenny et al. suggested that pulmonary particle deposition is determined by the residence time of the air in the peripheral airways and the fraction of the tidal volume that reaches the peripheral airways [35]. Actually, Rikard et al. showed that particle deposition in the nose and pharynx ranged from 17% to 19% in anesthetized piglets [36]. There may also be large amounts of particle deposition in the nose and pharynx in this study. The anesthetized animal model can be quite different from real clinical settings using inhalation. Therefore, the limitation of this study using an anesthetized animal must be considered in interpreting our data.

In this study, we attempted to visualize the ICG level of the lungs before complete systemic circulation occurred immediately after Exposure 2. ICG has several clinically excellent properties such as low toxicity, ideal for angiography, good SNR, deep imaging and simple imaging devices. ICG does not have any known metabolites, and it is extracted quickly by the liver into bile juice [37]. Despite well-established clinical use, understanding of the significant in vivo pharmacokinetics of systemically administered ICG was relatively limited by differences in solubility, dissolution and systemic uptake. Also, ICG should first be dissolved in water because ICG is not readily soluble in saline. Landsman et al. do not recommend adding isotonic saline and/or albumin to the injectate, when fast spectral stability is essential, for example, when using ICG for quantitative purposes [38]. Nevertheless, Kassab et al. investigated the aerosol particle size, output rate, extent of nebulization and stability during ICG nebulization, and they also measured the delivery and extracorporeal activation of the nebulized ICG in mice [27,28]. Thus, the result of ICG in Exposure 2 suggests that ICG can be reflected in pulmonary deposition as a tracer after nebulization.

The output rate of the nebulizer does not characterize the actual drug delivery to the patient. When investigating the drug delivery efficiency of the nebulizer in vitro and in vivo, the nebulizers were operated during the duration of nebulization. It is difficult to compare the drug delivery efficiencies because the output varies between different types of nebulizers. In order to compare salbutamol delivery efficiency, mice were exposed to salbutamol in Exposure 1 (duration of nebulization) and Exposure 2 (constant output 1.3 mL). In both exposure conditions, the salbutamol concentration in serum was higher in the SMN-U22 and VMN-SM1 groups than in the JN-PARIr group (Figure 2a,b). In addition, we showed that the ICG fluorescence signal intensities from the lung tissues and homogenates were higher in VMN-SM1 than in JN-PARIb. These results were similar to those of previous studies in which lung deposition was higher in mesh nebulizers than jet nebulizers in nebulization using 99mTc-DTPA alone or with salbutamol [18,19]. They directly showed lung scintigraphic images during or after the nebulization of 99mTc-DTPA. However, we showed that the salbutamol concentrations from lung tissues were no statistically significant differences, except in VMN-SM1 in Exposure 1. This is presumably due to the fact that salbutamol has already entered the blood through the lungs for 30 min after nebulization. The plasma salbutamol maximum concentration in humans appeared at 30–60 min after nebulization [39,40]. The higher levels of salbutamol in serum than in lung tissue means the drug has been systemically circulated through the lungs. Thus, the delivery efficiency of the mesh nebulizer can be expected to be better than that of the jet nebulizer.

Since Exposure 2 is the same output as 1.3 mL from the four nebulizers, the salbutamol delivery efficiency of each nebulizer in the lung and serum can be considered to be comparable. The salbutamol delivery efficiencies of SMN-U22 and VMN-SM1 were higher than that of JN-PARIr. The total amount of salbutamol delivered to lung and serum using SMN-U22 and VMN-SM1 was higher than 39.9% and 141.7% as compared to JN-PARIr, respectively. In addition, the ICG fluorescence signal intensities from lung tissues and homogenates were 60% and 58% higher in VMN-SM1 than JN-PARIb, respectively. Although the exposure times of the jet types were approximately 2–4 min longer than that of the mesh type in order to achieve the nebulization of 1.3 mL of salbutamol, it has little effect on salbutamol concentration in serum after 30 min. The delivery efficacy of the mesh nebulizer was higher than that of the jet nebulizer. This suggests that the use of a mesh nebulizer can reduce the dose of salbutamol present in clinical use. Coates et al. showed that the mesh nebulizer exhibits an equivalent level of tobramycin at lung deposition with a dose that is half of what is needed by the jet nebulizer [10]. While a jet nebulizer can generate smaller aerosol particles than the mesh nebulizer used in this study, the output rate is low, resulting in lower salbutamol delivery efficiency. This means that the output rate of the nebulizer plays an important role in salbutamol delivery efficiency in our exposure system. Finally, when using a mesh type nebulizer, care should be taken to avoid an overdose of salbutamol, because it has higher delivery efficiency than the jet nebulizer. Further studies are needed to demonstrate the effects of the output rate, exposure distance, and angle of nebulizer on drug delivery efficiency.

## Figures and Tables

**Table 1:** Nebulizers used in the study.

Mode of Operation	Model	Abbreviation in the Study
Jet	PARI BOY SX-red nozzle (PARI GmbH, Starnberg, Germany)	JN-PARIr
	PARI BOY SX-blue nozzle (PARI GmbH, Starnberg, Germany)	JN-PARIb
Static mesh	NE-U22 (Omron Healthcare, Kyoto, Japan)	SMN-U22
Vibrating mesh	NE-SM1 NEPLUS (KTMED Co., Seoul, Korea)	VMN-SM1

**Table 2:** Comparison of the aerosol particle sizes of four nebulizers tested with 1 and 1.25 mg/mL salbutamol.

Device	The Fifty Percent Volume Diameter (Dv(50) (μm))	
	1 mg/mL Salbutamol	1.25 mg/mL Salbutamol
JN-PARIr	3.40 ± 0.09	3.53 ± 0.05
JN-PARIb	4.54 ± 0.03	4.54 ± 0.01
SMN-U22	6.83 ± 0.09	6.86 ± 0.07
VMN-SM1	5.34 ± 0.18	5.51 ± 0.12

**Table 3:** Residual volumes, nebulization times, and output rates for four nebulizers.

Device	Residual Volume (mL)		Nebulization Time (min)		Output Rate (mL/min)	
	1 mg/mL Salbutamol	1.25 mg/mL Salbutamol	1 mg/mL Salbutamol	1.25 mg/mL Salbutamol	1 mg/mL Salbutamol	1.25 mg/mL Salbutamol
JN-PARIr	0.541 ± 0.021	0.536 ± 0.047	9.24 ± 0.05	9.33 ± 0.08	0.155 ± 0.002	0.153 ± 0.004
JN-PARIb	0.691 ± 0.011	0.684 ± 0.008	7.06 ± 0.05	7.10 ± 0.05	0.185 ± 0.002	0.184 ± 0.002
SMN-U22	0.039 ± 0.006	0.034 ± 0.008	6.48 ± 0.03	6.50 ± 0.09	0.288 ± 0.002	0.288 ± 0.006
VMN-SM1	0.041 ± 0.003	0.040 ± 0.002	8.16 ± 0.06	8.13 ± 0.06	0.237 ± 0.003	0.239 ± 0.003

**Table 4:** Nebulization time for output of 1.3 mL of salbutamol.

Device	Nebulization Time (min)
JN-PARIr	8.12 ± 0.007
JN-PARIb	7.05 ± 0.007
SMN-U22	4.34 ± 0.003
VMN-SM1	5.45 ± 0.003

## References

[1] Goralski J.L., Davis S.D. (2014). Breathing easier: Addressing the challenges of aerosolizing medications to infants and preschoolers. Respir. Med..

[2] Mansour M.M. (2013). Overcoming jet lag: Optimizing aerosol delivery with and without jet nebulizers. Respir. Care.

[3] Ari A. (2014). Jet, Ultrasonic, and Mesh Nebulizers: An Evaluation of Nebulizers for Better Clinical Outcomes. Eurasian J. Pulmonol..

[4] Ibrahim M., Verma R., Garcia-Contreras L. (2015). Inhalation drug delivery devices: Technology update. Med. Devices.

[5] Clay M.M., Pavia D., Newman S.P., Clarke S.W. (1983). Factors influencing the size distribution of aerosols from jet nebulisers. Thorax.

[6] Smith E.C., Denyer J., Kendrick A.H. (1995). Comparison of twenty three nebulizer/compressor combinations for domiciliary use. Eur. Respir. J..

[7] Kendrick A.H., Smith E.C., Denyer J. (1995). Nebulizers-fill volume, residual volume and matching of nebulizer to compressor. Respir. Med..

[8] Dhand R. (2002). Nebulizers that use a vibrating mesh or plate with multiple apertures to generate aerosol. Respir. Care.

[9] Waldrep J.C., Dhand R. (2008). Advanced nebulizer designs employing vibrating mesh/aperture plate technologies for aerosol generation. Curr. Drug Deliv..

[10] Coates A.L., Denk O., Leung K. (2011). Higher tobramycin concentration and vibrating mesh technology can shorten antibiotic treatment time in cystic fibrosis. Pediatr. Pulmonol..

[11] Michotte J.B., Jossen E., Roeseler J., Liistro G., Reychler G. (2014). In vitro comparison of five nebulizers during noninvasive ventilation: Analysis of inhaled and lost doses. J. Aerosol Med. Pulm. Drug Deliv..

[12] Vecellio L. (2006). The mesh nebuliser: A recent technical innovation for aerosol delivery. Breathe.

[13] Rabea H., Ali A.M., Salah Eldin R., Abdelrahman M.M., Said A., Abdelrahim M.E. (2017). Modelling of in-vitro and in-vivo performance of aerosol emitted from different vibrating mesh nebulisers in non-invasive ventilation circuit. Eur. J. Pharm. Sci..

[14] Ari A., Atalay O.T., Harwood R., Sheard M.M., Aljamhan E.A., Fink J.B. (2010). Influence of nebulizer type, position, and bias flow on aerosol drug delivery in simulated pediatric and adult lung models during mechanical ventilation. Respir. Care.

[15] Alhamad B.R., Fink J.B., Harwood R.J., Sheard M.M., Ari A. (2015). Effect of Aerosol Devices and Administration Techniques on Drug Delivery in a Simulated Spontaneously Breathing Pediatric Tracheostomy Model. Respir. Care.

[16] Ari A., de Andrade A.D., Sheard M., AIHamad B., Fink J.B. (2015). Performance Comparisons of Jet and Mesh Nebulizers Using Different Interfaces in Simulated Spontaneously Breathing Adults and Children. J. Aerosol Med. Pulm. Drug Deliv..

[17] Dugernier J., Hesse M., Jumetz T., Bialais E., Roeseler J., Depoortere V., Michotte J.B., Wittebole X., Ehrmann S., Laterre P.F. (2017). Aerosol Delivery with Two Nebulizers Through High-Flow Nasal Cannula: A Randomized Cross-Over Single-Photon Emission Computed Tomography-Computed Tomography Study. J. Aerosol Med. Pulm. Drug Deliv..

[18] Dubus J.C., Vecellio L., De Monte M., Fink J.B., Grimbert D., Montharu J., Valat C., Behan N., Diot P. (2005). Aerosol deposition in neonatal ventilation. Pediatr. Res..

[19] Galindo-Filho V.C., Ramos M.E., Rattes C.S., Barbosa A.K., Brandão D.C., Brandão S.C., Fink J.B., de Andrade A.D. (2015). Radioaerosol Pulmonary Deposition Using Mesh and Jet Nebulizers During Noninvasive Ventilation in Healthy Subjects. Respir. Care.

[20] Murayama N., Murayama K. (2018). Comparison of the Clinical Efficacy of Salbutamol with Jet and Mesh Nebulizers in Asthmatic Children. Pulm. Med..

[21] Dunne R.B., Shortt S. (2018). Comparison of bronchodilator administration with vibrating mesh nebulizer and standard jet nebulizer in the emergency department. Am. J. Emerg. Med..

[22] Song X., Hu J., Zhan S., Zhang R., Tan W. (2016). Effects of Temperature and Humidity on Laser Diffraction Measurements to Jet Nebulizer and Comparison with NGI. AAPS PharmSciTech.

[23] Technical Committee ISO/TC 121/SC 2 (2013). ISO 27427:2013, Anaesthetic and respiratory equipment-Nebulizing Systems and Components.

[24] Tandon R., McPeck M., Smaldone G.C. (1997). Measuring Nebulizer Output Aerosol Production vs Gravimetric Analysis. Chest.

[25] Sevick-Muraca E.M. (2012). Translation of near-infrared fluorescence imaging technologies: Emerging clinical applications. Annu. Rev. Med..

[26] Marshall M.V., Rasmussen J.C., Tan I.C., Aldrich M.B., Adams K.E., Wang X., Fife C.E., Maus E.A., Smith L.A., Sevick-Muraca E.M. (2010). Near-infrared fluorescence imaging in humans with indocyanine green: A review and update. Open Surg. Oncol. J..

[27] Kassab G., Geralde M.C., Inada N.M., Achiles A.E., Guerra V.G., Bagnato V.S. (2019). Nebulization as a tool for photosensitizer delivery to the respiratory tract. J. Biophotonics.

[28] Kassab G., Geralde M.C., Inada N.M., Bagnato V.S. (2018). Fluorescence assessment of the delivery and distribution of nebulized. Proceedings of the Photo-Optical Instrumentation Engineers (SPIE).

[29] Usmani O.S., Biddiscombe M.F., Barnes P.J. (2005). Regional lung deposition and bronchodilator response as a function of beta2-agonist particle size. Am. J. Respir. Crit. Care Med..

[30] Khilnani G.C., Banga A. (2008). Aerosol therapy. Indian J. Chest Dis. Allied Sci..

[31] Nikander K., Denyer J., Everard M., Smaldone G.C. (2000). Validation of a new breathing simulator generating and measuring inhaled aerosol with adult breathing patterns. J. Aerosol Med..

[32] Nikander K., Denyer J., Smith N., Wollmer P. (2001). Breathing patterns and aerosol delivery: Impact of regular human patterns, and sine and square waveforms on rate of delivery. J. Aerosol Med..

[33] Sangwan S., Condos R., Smaldone G.C. (2003). Lung deposition and respirable mass during wet nebulization. J. Aerosol Med..

[34] Bosco A.P., Rhem R.G., Dolovich M.B. (2005). In vitro estimations of in vivo jet nebulizer efficiency using actual and simulated tidal breathing patterns. J. Aerosol Med..

[35] Rissler J., Gudmundsson A., Nicklasson H., Swietlicki E., Wollmer P., Löndahl J. (2017). Deposition efficiency of inhaled particles (15-5000 nm) related to breathing pattern and lung function: An experimental study in healthy children and adults. Part. Fibre Toxicol..

[36] Linner R., Perez-de-Sa V., Cunha-Goncalves D. (2015). Lung deposition of nebulized surfactant in newborn piglets. Neonatology.

[37] Alander J.T., Kaartinen I., Laakso A., Pätilä T., Spillmann T., Tuchin V.V., Venermo M., Välisuo P. (2012). A review of indocyanine green fluorescent imaging in surgery. Int. J. Biomed. Imaging.

[38] Landsman M.L., Kwant G., Mook G.A., Zijlstra W.G. (1976). Light-absorbing properties, stability, and spectral stabilization of indocyanine green. J. Appl. Physiol..

[39] Gawchik S.M., Saccar C.L., Noonan M., Reasner D.S., DeGraw S.S. (1999). The safety and efficacy of nebulized levalbuterol compared with racemic albuterol and placebo in the treatment of asthma in pediatric patients. J. Allergy Clin. Immunol..

[40] Usmani O.S., Biddiscombe M.F., Yang S., Meah S., Oballa E., Simpson J.K., Fahy W.A., Marshall R.P., Lukey P.T., Maher T.M. (2018). The topical study of inhaled drug (salbutamol) delivery in idiopathic pulmonary fibrosis. Respir. Res..

